# Effects of waterlogging on microbial activity, soil nutrient availability, nutrient uptake, and yield of tolerant and sensitive onion genotypes

**DOI:** 10.3389/fpls.2025.1692450

**Published:** 2025-11-13

**Authors:** Amol R. Pawar, Sushant Sukumar Patil, Mayur B. Patil, Payal A. Mahadule, Komal Anil Gade, Thangasamy Arunachalam, Vijay B. Mahajan

**Affiliations:** 1 Department of Agronomy, School of Agriculture, Lovely Professional University, Phagwara, Punjab, India; 2 Department of Soil Science, Indian Council of Agricultural Research (ICAR)-Directorate of Onion and Garlic Research Pune, Maharashtra, India

**Keywords:** monsoon onion, tolerant genotypes, Bhima Dark Red, Accession 1666, dehydrogenase, redundancy analysis

## Abstract

**Introduction:**

Rainfall variability during the monsoon season poses a major challenge to onion production, especially due to waterlogging stress in clay loam soils. Saturated conditions reduce soil aeration, disrupt microbial activity and nutrient transformations, and impair nutrient uptake and crop performance.

**Methods:**

To investigate these effects, a field experiment was conducted under a split-plot design with flatbed layout to assess changes in soil physical properties, microbial activity, nutrient availability, and their combined effects on nutrient uptake and bulb yield in eight onion genotypes (two tolerant and six sensitive).

**Results and Discussion:**

Waterlogging increased bulk density by 5.30% and reduced infiltration rate by 76.5% compared to control. At 50 days after transplanting (DAT), microbial biomass carbon declined by 67.6%, while dehydrogenase, urease, acid phosphatase, and alkaline phosphatase activities declined by 55.8%, 33.9%, 33.9%, and 10.2%, respectively. Available macronutrients (N, P, K, S, Ca, Mg) and micronutrients (Fe, Cu, Mn, B) were significantly reduced at 55 DAT compared to 45 DAT. These changes led to reduced nutrient uptake and yield across genotypes. However, tolerant genotypes Accession 1666 and Bhima Dark Red (BDR) Selection exhibited better tolerance, with only 21.7% and 18.1% yield reductions, compared to 41.6–64.8% in sensitive types. Raised bed planting further improved performance of tolerant genotypes under waterlogged conditions.

**Conclusion:**

These findings highlight genotypic selection and raised-bed cultivation as effective strategies to mitigate waterlogging stress in monsoonal onion systems.

## Introduction

India contributes nearly 20% of global onion production, with the Deccan Plateau accounting for 50% of the national output. Onions are cultivated in three seasons—monsoon, late monsoon, and winter. Of these, monsoon and late monsoon crops are more severely affected by waterlogging compared to winter crops (Gedam et al., 2022; Thangasamy et al., 2023), resulting in market instability and frequent price fluctuations (Ghodke et al., 2018; Gedam et al., 2023). The increasing frequency and intensity of rainfall due to climate change have further exacerbated waterlogging issues, limiting onion productivity (Thangasamy et al., 2023). The shallow root system of onions makes them particularly susceptible to waterlogged conditions (Ghodke et al., 2018). Yield losses under moderate waterlogging range from 25–30%, while severe waterlogging can result in complete crop failure (Dubey et al., 2021). Gedam et al. (2022) reported that excessive rainfall could reduce bulb yield by 50–70%, posing a major challenge to onion cultivation during monsoon, especially with increasingly erratic monsoon patterns and unseasonal rains.

In India, floods affect an average of 7.5 million hectares of agricultural land annually (Sarkar, 2022), and from 2015 to 2021, extreme weather events such as floods have damaged approximately 33.9 million hectares of cropped area. Waterlogging has significant implications for soil health and nutrient dynamics (Pais et al., 2023). It increases bulk density and reduces total pore volume, thereby affecting water-holding capacity (Todisco et al., 2022). Saturated soil conditions reduce aeration, alter gas exchange and composition, and suppress microbial and plant growth (Wang et al., 2025). Additionally, particle dispersion under waterlogged conditions leads to surface crusting and reduced infiltration (Awedat et al., 2021). The resultant oxygen deficiency in the root zone triggers a shift from aerobic to anaerobic processes, thereby altering the soil’s redox potential and influencing nutrient transformation and availability (Ben-Noah and Friedman, 2018).

This anaerobic condition shift in soil conditions affects the solubility and availability of essential nutrients. Waterlogging increases the availability of iron (Fe) and manganese (Mn), potentially leading to toxicity, while reducing nitrogen (N) availability through denitrification and leaching (Tyagi et al., 2024). Anaerobic conditions can temporarily increase phosphorus (P) availability due to the reduction of Fe and Mn oxides (Salunkhe et al., 2022), while prolonged waterlogging often results in P deficiency due to restricted root growth and nutrient uptake (Yang et al., 2024).

Soil enzymatic activities serve as important indicators of soil fertility and health (Wang et al., 2023). Waterlogging significantly affects enzymes such as dehydrogenase, urease, alkaline phosphatase, and acid phosphatase (Gu et al., 2019), all of which play key roles in nutrient cycling and organic matter decomposition (Daunoras et al., 2024). Moreover, waterlogging leads to major shifts in the microbial community—declining aerobic populations and increasing anaerobic and facultative anaerobic organisms (Randle‐Boggis et al., 2018), with profound implications for soil nutrient cycling and overall health (Salunkhe et al., 2022). Oxygen deprivation in the root zone impairs root function, nutrient, and water uptake (Gedam et al., 2022), contributing to nutrient stress that ultimately affects onion bulb yield and quality (Salunkhe et al., 2022; Gedam et al., 2023).

Despite a general understanding of plant responses to waterlogging, limited studies have explored how waterlogging affects soil physical properties—particularly infiltration rate and bulk density—as well as nutrient availability and microbial activities in onion crops. Understanding these changes is essential for developing strategies to improve resilience under such conditions. Therefore, the present study was formulated with the hypotheses: 1. Waterlogging will increase bulk density while decreasing infiltration rate, microbial activity, nutrient transformation, and nutrient uptake. 2. Reduced nutrient availability and uptake will negatively affect plant growth, bulb size, and yield in onion genotypes. To test these hypotheses, the present study was undertaken to assess the effects of waterlogging on infiltration rate, bulk density, nutrient availability, and microbial activities. The study also evaluated how these changes influenced nutrient uptake, plant growth, and bulb yield in onion genotypes under waterlogged conditions. This study integrates assessments of soil physical properties, microbial activities, and nutrient availability to better understand the responses of onion genotypes to waterlogging stress.

## Materials and methods

### Experimental site

The field experiment was conducted at the Indian Council of Agricultural Research–Directorate of Onion and Garlic Research (ICAR–DOGR) experimental farm in Pune, Maharashtra, India (18.32° N, 73.51° E, 645 MSL) during the monsoon season (August to November 2023). The site experiences a tropical dry and humid climate. It receives an average annual rainfall of 820 mm, with approximately 99% occurring during the southwest monsoon season (June to October). The mean temperature during the experimental period ranged from 9.7 °C to 41.2 °C. The soil at the experimental site was classified as *Typic Haplustepts*, characterized as clay loam with a pH of 7.63, electrical conductivity of 0.25 dS m^-^¹, and soil organic carbon content of 8.6 mg kg^-^¹. The soil is low in N while high in P and K and contains adequate levels of S and micronutrients.

### Experimental details

A split-plot design was adopted with eight onion genotypes evaluated under two conditions: control (well-watered) and waterlogged. The genotypes included four sensitive (Bhima Super, Bhima Shubra, Bhima Red, and Bhima Raj) and four tolerant (Accession 1666, Accession 1630, Bhima Dark red (BDR) Selection, and W 355) genotypes. Each treatment was replicated three times. Flat beds measuring 2 m × 3 m were prepared, and well-decomposed farmyard manure was incorporated at 5 t ha^-^¹. A basal dose comprising 16% of the recommended N, along with full doses of P, K, and S, was applied before transplanting. The nutrient sources included complex fertilizer (10:26:26), urea, muriate of potash, and bentonite sulfur. Pre-emergence herbicide oxyfluorfen (23% emulsifiable concentrates) was applied 15 minutes before transplanting, followed by irrigation to ensure uniform distribution. Forty-five-day-old seedlings were transplanted at 15 cm × 10 cm spacing, with a population of 400 plants per plot. Both control and waterlogged plots were irrigated uniformly until 45 days after transplanting (DAT). Waterlogging treatment was initiated at 45 DAT and continued for 10 days until 55 DAT. During this period, daily flooding in the morning was followed by sprinkler irrigation until 6:00 PM to simulate rainfall and maintain waterlogged conditions. After 55 DAT, normal irrigation resumed in all plots until harvest.

All agronomic practices and plant protection measures were followed as per ICAR–DOGR recommendations, particularly for managing anthracnose disease. Onion bulbs were harvested during the first week of November 2023. Equatorial and polar diameters of bulbs were measured using a vernier caliper and expressed in millimeters. Bulbs were separated from the foliage, and their weight was recorded and expressed in kg per plot. Plant height and number of leaves were recorded from ten tagged plants per plot at 45, 55, 75, and 90 DAT. Leaf area was measured using the third fully developed leaf from the top during the same intervals.

A parallel experiment using the same set of genotypes was conducted on raised beds, following identical agronomic practices as the flatbed system. During the waterlogging period, soil in the raised bed system remained saturated throughout the day without surface inundation. Yield parameters were recorded at harvest.

### Soil physical properties

Infiltration rate and bulk density were measured in both the waterlogged and control plots after the onion harvest. The soil infiltration rate was determined using the double-ring infiltrometer method (Bouwer, 1986). Bulk density was measured using the core method (Blake and Hartge, 1986).

### Soil enzymatic activity

Moist soil samples collected from each treatment were used to assess enzyme activities. Dehydrogenase activity was measured by the triphenyl tetrazolium chloride reduction method and expressed as µg triphenyl formazan (TPF) g^-^¹ dry soil h^-^¹ (Casida et al., 1964). Urease activity was determined by incubating 5 g of soil with 5 mL of 10 mg urea solution at 37 °C for 5 hours, followed by colorimetric estimation at 527 nm using diacetyl monoxime–thiosemicarbazide (DAM-TSC) reagents (Bremner and Douglas, 1971). Acid and alkaline phosphatase activities were determined by incubating 1 g of soil with p-nitrophenyl phosphate substrate in pH-adjusted buffers (pH 6.5 for acid and pH 11 for alkaline phosphatase) at 37 °C for 1 hour. Reactions were stopped with CaCl_2_-NaOH solution, and p-nitrophenol released was measured at 400 nm (Tabatabai and Bremner, 1969). Soil microbial biomass carbon (SMBC) was estimated using the chloroform fumigation-extraction method (Jenkinson and Powlson, 1976). Fresh soil (10 g) was fumigated with ethanol-free chloroform for 24 hours in a vacuum desiccator. Both fumigated and unfumigated samples were extracted with 0.5 M K_2_SO_4_, filtered, and analyzed for organic carbon via dichromate oxidation. Results were expressed as µg C g^-^¹ dry soil.

### Soil sampling and analysis

Soil samples were collected from a 0–15 cm depth at five time points: pre-planting, 45, 55, and 75 days after transplanting (DAT), and at harvest, using a post-hole auger. Samples were collected between plant rows, air-dried, sieved (2 mm), and analyzed. Soil pH and electrical conductivity were measured using pH meter and conductivity bridge. Soil organic carbon (SOC) was estimated using the Walkley and Black (1934) wet oxidation method. Available N was analyzed using the alkaline permanganate method (Subbiah and Asija, 1956), P by the Olsen method (Olsen et al., 1954), and K using 1N ammonium acetate extraction (Jackson, 1967). Available S was extracted with 0.15% CaCl_2_ and analyzed using the turbidimetric method (Chesnin and Yien, 1951). Ca and Mg were determined by EDTA titration (Jackson, 1967), and micronutrients via atomic absorption spectrophotometry. DTPA extractable Fe, Mn, Zn, and Cu were extracted and analyzed in an atomic absorption spectrometer (Lindsay and Norvell, 1978). Available boron was extracted using hot water and determined in a spectrophotometer using the azomethine-H method (John et al., 1975).

### Plant sampling and analysis

Whole plant samples (five per treatment) were collected at 45 DAT, 55 DAT, and at harvest. Samples were washed, rinsed with distilled water, and separated into bulbs and leaves. They were chopped, air-dried, and subsequently oven-dried at 60 °C until a constant weight was reached. The dry weight of bulbs and leaves was recorded, and the samples were ground and sieved (2.0 mm) for nutrient analysis. Total N was determined using the micro-Kjeldahl method. For P, K, and S estimation, 0.5 g of the ground sample was digested using a di-acid mixture, filtered through Whatman No. 40 paper, and analyzed. P was measured using the ammonium vanado-molybdate method, K with a flame photometer, and S via the turbidimetric method (Jackson, 1967). Ca and Mg concentrations were estimated using the modified EDTA titration method, while micronutrients (Fe, Zn, Mn, Cu) were analyzed using atomic absorption spectrophotometry (Lindsay and Norvell, 1978). Nutrient uptake was calculated by multiplying nutrient concentration with dry matter yield and expressed as kg ha^-^¹ for macronutrients and g ha^-^¹ for micronutrients.

### Statistical analysis

The field experiment was conducted in a split-plot design with two treatments (control and waterlogging) as main plots and genotypes as sub-plots, replicated four times. Data were analyzed using Python (version 3.11; Python Software Foundation, 2023). Two-way ANOVA and mixed-model analyses were performed using the statsmodels library, considering treatment as a fixed factor and replication as a random effect. Treatment means were compared using the Tukey–Kramer Honestly Significant Difference (HSD) test at *p* ≤ 0.05 to identify significant differences among treatments and genotypes. Infiltration rate and bulk density data were analyzed using an independent two-sample *t*-test (*p* < 0.05, two-tailed).

Multivariate analyses were conducted to assess the interrelationships among soil physical, biological, and chemical parameters, nutrient uptake, and yield under control and waterlogged conditions. The first redundancy analysis (RDA) was performed using the scikit-learn library, incorporating soil physical properties (bulk density, infiltration rate), soil microbial activities (dehydrogenase enzyme, soil microbial biomass carbon, and urease activity), and available nutrient parameters as explanatory variables, with nutrient uptake and yield as response variables (*n* = 8). This analysis aimed to identify indicative relationships between soil conditions and crop responses under waterlogging, acknowledging the limitations associated with a small sample size. The second RDA was conducted using nutrient uptake and yield data (*n* = 64) to examine the direct associations between nutrient uptake efficiency and productivity under waterlogging stress. RDA biplots were generated using the matplotlib package in Python to illustrate the separation between control and waterlogged treatments.

Pearson’s correlation analysis (two-tailed) was also performed to examine linear associations among soil, microbial, nutrient uptake, and yield traits. Additionally, tolerance level, stress tolerance index, and stress sustainability index were computed to quantify genotype performance under waterlogged and control conditions, using standard equations based on yield under waterlogging stress and the control.

## Results

### Soil physical and microbial properties

**Figure 1:**
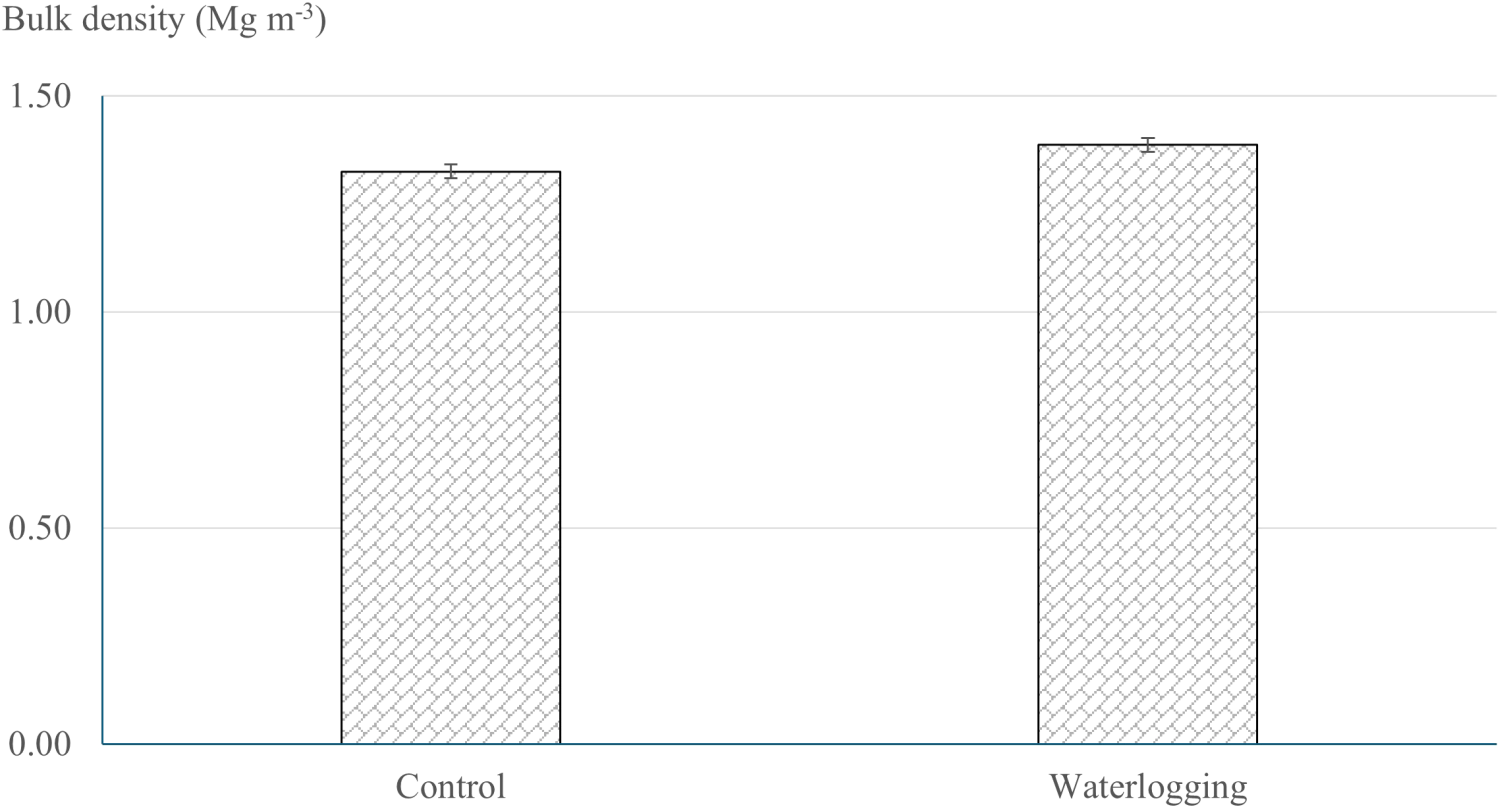
Effect of waterlogging on soil bulk density. Soil bulk density was measured at harvest under control and waterlogged conditions. Values represent mean ± SE.

**Figure 2:**
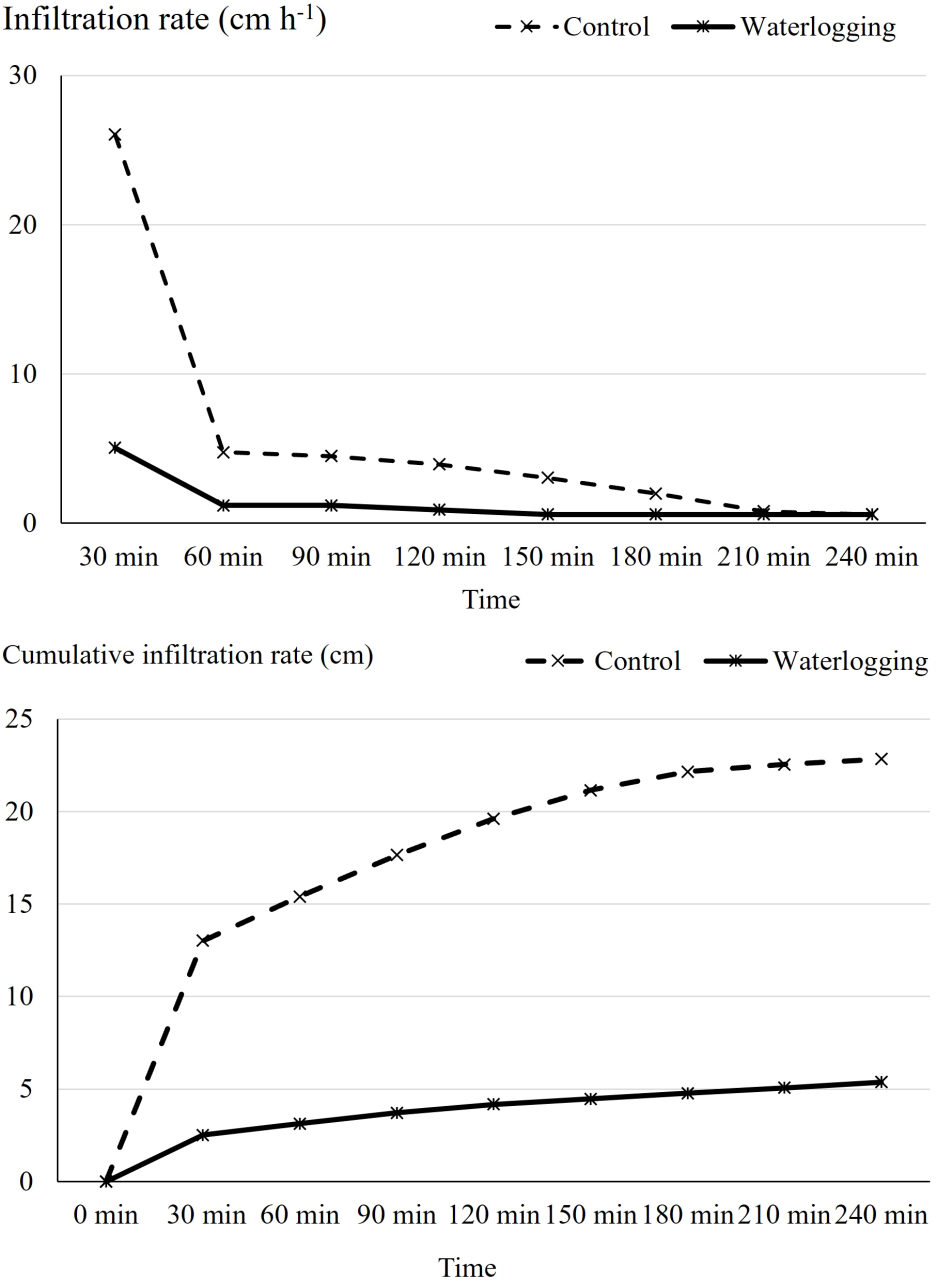
Effect of waterlogging on cumulative infiltration and infiltration rate. Cumulative infiltration and infiltration rate were assessed at harvest under control and waterlogged conditions. Values represent mean ± SE.

Infiltration rate and bulk density exhibited significant differences between the waterlogged and control plots. Waterlogging significantly increased soil bulk density from 1.32 Mg m^-^³ in the control to 1.39 Mg m^-^³ (p = 0.001), representing a 5.30% increase compared to the control plots (**Figure 1**). Waterlogging treatments recorded significantly lower infiltration rate and cumulative infiltration compared to the control (p < 0.0001). Cumulative infiltration under waterlogged conditions was reduced by 76.5% relative to the control (**Figure 2**). Moreover, infiltration rate stabilized within one hour in the waterlogged plots, whereas it continued to increase and stabilized only after four hours in the control (**Figure 2**). Additionally, waterlogging significantly influenced SMBC, dehydrogenase, urease, and both acid and alkaline phosphatase activities across growth stages (**Table 1**). At 50 DAT, waterlogged conditions led to reductions of 67.6% in SMBC, 55.8% in dehydrogenase, 33.9% in urease, 33.9% in acid phosphatase, and 10.2% in alkaline phosphatase compared to their respective values recorded at 45 DAT (pre-treatment). Post waterlogging stress at 55 DAT, SMBC, acid phosphatase, and alkaline phosphatase activities recovered slightly, showing reductions of 25.0%, 12.3%, and 12.3%, respectively, relative to their pre-treatment levels. Notably, dehydrogenase and urease activities at 55 DAT remained statistically similar to the levels observed at 50 DAT, indicating minimal recovery. In contrast, the control plots showed only minor changes in soil microbial biomass carbon, dehydrogenase, urease, and acid and alkaline phosphatase activities at 50 and 55 DAT compared to their respective pre-treatment values. Bulk density showed a significant negative correlation with soil microbial activities, infiltration rate, soil available nutrients, nutrient uptake, and bulb yield, whereas cumulative infiltration exhibited a significant positive correlation with these parameters (**Supplementary Table 1**). Soil enzyme activities—dehydrogenase, acid phosphatase, and urease—also showed significant positive correlations with nutrient availability, nutrient uptake, and bulb yield.

**Table 1:** Effect of waterlogging on SMBC, and soil enzymatic activity at pre- and post-waterlogging stages.

Days after transplanting	SMBC (μg g^-1^ soil)		Dehydrogenase (μg TPF g^-^¹ h^-^¹)		Acid-phosphatase (μg PNP g^-^¹ h^-^¹)		Alkaline-phosphatase (μg PNP g^-^¹ h^-^¹)		Urease (μg NH4-N g^-1^ soil h^-1^)	
	Control	Water-logging	Control	Water-logging	Control	Water-logging	Control	Water-logging	Control	Water-logging
45 DAT	62.8	62.9	46.6	46.4	97.2	86.3	97.2	97.5	73.1	72.5
50 DAT	52.9	20.4	45.1	20.5	99.6	63.7	102.6	64.5	85.9	65.1
55 DAT	57.7	47.2	42.9	19.5	101.0	101.0	111.6	85.5	90.7	66.9
Factors	Tukey–Kramer HSD values (p<0.05)									
Stages	5.9		4.7		8.7		9.1		5.2	
Waterlogging	4.3		3.5		6.4		7.0		3.9	
Stages × Waterlogging	7.3		6.1		10.9		11.1		6.9	

DAT, Days after transplanting; HSD, Honestly significant difference; SMBC, Soil microbial biomass carbon.

### Soil nutrient available status

Treatments, waterlogging stages, and their interactions significantly affected soil-available macro- (**Table 2**) and micronutrient concentrations (**Table 3**). At 55 DAT, waterlogging markedly reduced the concentrations of available N, P, K, S, Ca, Mg, Fe, Cu, Mn, and B compared to their respective pre-treatment values recorded at 45 DAT. Specifically, the reductions were 33.4% for N, 27.8% for P, 16.5% for K, 22.7% for S, 12.5% for Ca, and 35.9% for Mg. Among the micronutrients, Fe, Mn, Cu, and B decreased by 31.8%, 32.9%, 22.1%, and 34.0%, respectively, compared to 45 DAT. Notably, the concentration of available Zn increased by 68.9% at 55 DAT relative to its pre-treatment level. During the recovery period at 75 DAT, nutrient availability improved compared to 55 DAT. Despite this, most nutrient concentrations remained substantially lower than the pre-treatment levels recorded at 45 DAT. In contrast, available Zn and B levels at 75 DAT were 65.6% and 27.7% higher, respectively, than their pre-treatment values. Meanwhile, the available nutrient concentrations in the control plots showed little change throughout the sampling period compared to the pre-treatment values. Waterlogging also led to reductions in soil pH, EC, and SOC by 7.9%, 8.0%, and 3.2%, respectively, compared to pre-treatment levels. By 75 DAT, soil pH, EC, and SOC had increased by 10.0%, 4.3%, and 30.6%, respectively, compared to their values at 55 DAT, indicating partial recovery of soil chemical properties. Soil available nutrients, both macro- and micronutrients (except zinc), showed significant positive correlations with nutrient uptake and bulb yield.

**Table 2:** Effect of waterlogging on soil available macronutrient availability at pre- and post-harvest stages.

Days after transplanting	N (mg kg^-1^)		P (mg kg^-1^)		K (mg kg^-1^)		S (mg kg^-1^)		Ca (cmol (p^+^) kg^-1^)		Mg (cmol (p^+^) kg^-1^)	
	Control	Water-logging	Control	Water-logging	Control	Water-logging	Control	Water-logging	Control	Water-logging	Control	Water-logging
Initial	93.3	89.6	21.0	21.1	270.2	269.3	10.1	9.9	1.10	1.08	0.53	0.48
45 DAT	99.3	98.2	21.5	21.9	370.4	367.7	17.2	17.1	1.18	1.17	0.67	0.64
55 DAT	104.6	65.4	20.8	15.8	378.0	306.8	16.3	13.3	1.40	1.02	0.80	0.41
75 DAT	122.5	74.7	22.2	17.3	400.8	319.8	17.4	13.9	1.48	1.15	1.03	0.54
Harvest	85.8	70.9	17.6	15.0	354.0	297.9	14.0	13.0	1.02	0.97	0.52	0.41
Factors	Tukey–Kramer HSD values (p<0.05)											
Stage	9.9		1.5		38.6		1.4		0.14		0.19	
Waterlogging	7.3		1.1		29.7		1.1		0.11		0.15	
Stage × Waterlogging	13.0		2.0		52.1		2.1		0.19		0.24	

HSD, Honestly significant difference; DAT, Days after transplanting; N, Nitrogen; P, Phosphorus; K, Potassium; S, Sulphur; Ca, Calcium; Mg, Magnesium.

**Table 3:** Effect of waterlogging on soil micronutrient availability at pre- and post-waterlogging stages.

Treatment/DAT	Fe (mg kg^-1^)		Mn (mg kg^-1^)		Zn (mg kg^-1^)		Cu (mg kg^-1^)		B (mg kg^-1^)	
	Control	Water-logging	Control	Water-logging	Control	Water-logging	Control	Water-logging	Control	Water-logging
Initial	2.87	2.87	4.00	3.93	1.00	1.13	4.00	4.00	0.84	0.84
45 DAT	3.97	4.00	5.13	5.13	1.76	1.80	6.02	6.07	0.92	0.94
55 DAT	4.40	2.73	5.67	3.44	3.14	3.04	5.70	4.73	0.92	0.62
75 DAT	4.67	3.55	6.07	4.25	3.47	2.98	6.92	5.49	1.18	1.20
At harvest	3.63	2.78	4.84	4.07	5.13	2.89	5.93	5.24	0.87	0.89
Factors	Tukey–Kramer HSD values (p<0.05)									
Stage	0.48		0.52		0.66		0.69		0.09	
Waterlogging	0.34		0.39		0.45		0.52		0.07	
Stage × Waterlogging	0.63		0.66		0.78		0.83		0.12	

HSD, Honestly significant difference; DAT, Days after transplanting; Fe, Iron; Mn, Manganese; Zn, Zinc; Cu, Copper; B, Boron.

### Total nutrient uptake

Genotypes, waterlogging, and their interaction significantly influenced both macro- (**Table 4**) and micronutrient uptake (**Table 5**). Under waterlogged conditions, Accession 1666 and BDR selection exhibited comparatively lower reductions in nutrient uptake than the other genotypes, relative to their respective control plants. Accession 1666 showed reductions of 19.8% in N, 13.8% in P, 20.4% in K, and 18.3% in S. For micronutrients, this genotype recorded decreases of 25.2% in Fe, 45.7% in Mn, 31.5% in Zn, 33.8% in Cu, and 25.5% in B. Similarly, BDR Selection exhibited reductions of 14.1% in N, 11.4% in P, 32.7% in K, and 35.4% in S. Its micronutrient uptake declined by 26.6% for Fe, 57.1% for Mn, 26.7% for Zn, 35.4% for Cu, and 24.4% for B. In contrast, the sensitive genotypes experienced markedly higher reductions in nutrient uptake under waterlogging stress. Their uptake of macronutrients decreased by 41.6%–65.5% for N, 43.1%–70.2% for P, 35.3%–61.7% for K, and 51.1%–70.8% for S. Micronutrient uptake in these genotypes declined by 41.5%–57.6% for Fe and 62.3%–77.6% for Mn. Additionally, Zn, Cu, and B uptake decreased by 47.2%–66.8%, 52.3%–72.7%, and 42.4%–72.7%, respectively, compared to their control plants.

**Table 4:** Effect of waterlogging on major nutrient uptake in onion genotypes at harvest.

Genotypes	Total N uptake (kg ha^−1^)		Total P uptake (kg ha^−1^)			Total K uptake (kg ha^−1^)		Total S uptake (kg ha^−1^)		Total Ca uptake (kg ha^−1^)		Total Mg uptake (kg ha^−1^)	
	Control	Water-logging	Control	Water-logging	Control		Water-logging	Control	Water-logging	Control	Water-logging	Control	Water-logging
Accession 1666	97.17	77.96	16.67	14.37	96.09		76.50	24.04	19.62	50.32	37.14	11.95	8.78
Accession 1630	90.16	52.65	16.04	9.08	90.93		56.02	28.43	13.88	45.52	23.13	11.00	5.36
W 355	79.46	36.53	12.36	6.30	90.39		42.58	25.42	9.40	34.58	16.62	10.09	5.23
BDR Selection	83.13	71.38	13.19	11.67	101.67		68.39	27.11	17.45	46.56	31.72	12.85	8.94
Bhima Red	44.89	23.34	5.93	3.18	52.39		33.88	11.77	4.90	24.70	13.35	6.53	2.25
Bhima Raj	85.61	36.50	11.92	5.47	76.90		41.84	22.30	9.32	40.06	18.56	10.59	3.10
Bhima Shubra	69.20	23.85	9.70	3.48	72.06		27.63	19.93	6.17	34.30	11.79	10.22	2.43
Bhima Super	62.87	23.78	11.36	3.36	62.04		29.11	17.79	5.22	31.05	11.84	9.08	2.84
Factors	Tukey–Kramer HSD values (p<0.05)												
Waterlogging (W)	15.17		7.82			14.51		5.07		4.03		0.93	
Genotype (G)	7.95		4.95			8.02		2.28		4.54		1.05	
W×G	12.88		8.02			13.01		3.69		7.37		1.70	

HSD, Honestly significant difference.

**Table 5:** Effect of waterlogging on micronutrient uptake in onion genotypes at harvest.

Genotypes	Total Fe uptake (kg ha^−1^)		Total Mn uptake (kg ha^−1^)		Total Zn uptake (kg ha^−1^)		Total Cu uptake (kg ha^−1^)		Total B uptake (kg ha^−1^)	
	Control	Water-logging	Control	Water-logging	Control	Water-logging	Control	Water-logging	Control	Water-logging
Accession 1666	1124.4	841.6	272.2	147.8	272.8	186.8	105.4	69.8	222.8	166.0
Accession 1630	1048.3	613.2	245.4	84.8	265.6	126.1	97.0	46.3	240.8	138.8
W 355	897.3	479.0	202.2	55.5	208.3	93.3	83.0	32.4	202.7	113.0
BDR Selection	1035.3	759.9	252.4	108.2	233.3	171.0	96.1	62.1	231.7	175.1
Bhima Red	566.3	356.8	142.2	53.7	112.0	59.1	49.8	22.0	118.1	51.0
Bhima Raj	916.5	470.6	228.3	71.9	190.9	81.9	83.7	29.6	191.2	82.4
Bhima Shubra	757.4	321.5	193.6	43.4	142.6	47.3	69.9	19.2	176.2	48.1
Bhima Super	682.9	341.8	174.1	46.4	124.5	50.0	64.3	20.1	166.4	52.7
Factors	Tukey–Kramer HSD values (p<0.05)									
Waterlogging (W)	81.3		17.8		18.5		6.2		25.1	
Genotype (G)	91.8		20.1		20.9		7.0		28.4	
W×G	148.9		32.7		32.7		11.4		46.0	

HSD, Honestly significant difference.

### Plant growth parameters

Waterlogging, genotypes, and their interaction significantly influenced plant growth parameters, including plant height (**Supplementary Table 3**), number of leaves (**Supplementary Table 4**), and total leaf area (**Supplementary Table 5**) across all growth stages. Under waterlogged conditions, Accession 1666 exhibited reductions of 7.2% in plant height, 22.7% in number of leaves, and 3.8% in total leaf area at 55 DAT compared to its control plants. BDR Selection showed slightly higher reductions, with plant height, number of leaves, and total leaf area declining by 22.2%, 21.9%, and 6.6%, respectively. Accession 1630 and W-355 also demonstrated relatively lesser reductions than those observed in the sensitive genotypes. In contrast, the sensitive genotypes under waterlogging stress showed significantly higher reductions, with plant height reduced by 31.4%–38.9%, number of leaves by 51.5%–58.3%, and total leaf area by 55.4%–60.8% compared to their respective control plants. All genotypes showed signs of recovery after the cessation of waterlogging, with improvements observed in plant height, leaf number, and leaf area by 75 DAT. By 90 DAT, tolerant genotypes (Accession 1666, BDR Selection, Accession 1630, and W-355) showed a slight decline in leaf number and area, indicating a slowdown in vegetative growth. By comparison, the sensitive genotypes continued to recover, with increases of 118.8%–122.6% in leaf number and 120.4%–147.7% in leaf area compared to 55 DAT.

### Bulb size and yield

Both waterlogging and genotypes significantly affected bulb size, marketable bulb yield, and total bulb yield, whereas their interaction was not significant under either flatbed or raised bed conditions (**Table 6**). Tolerant genotypes such as Accession 1666 and BDR Selection experienced comparatively lower reductions in bulb size, yield, and dry matter under waterlogged conditions. Among these, Accession 1666 showed the least reduction, with a 21.7% decrease in bulb yield and minor reductions in equatorial (16.7%) and polar (4.8%) bulb diameters. BDR Selection also exhibited moderate losses in bulb yield (18.1%) and associated parameters. Accession 1666 showed a high stress tolerance index (STI) (1.03) and low stress susceptibility index (SSI) (<1), indicating strong stability under stress, whereas BDR Selection achieved the highest STI (1.20) and exhibited a slightly higher SSI (>1) (**Table 6**). Other genotypes recorded the lowest stressed yields and highest susceptibility indices, confirming their sensitivity to waterlogging under both flatbed and raised bed system. Additionally, a parallel experiment using a raised bed system with sprinkler irrigation to simulate rainfall resulted in higher marketable bulb yields in the tolerant genotypes, Accession 1666 (16.0 t/ha) and BDR Selection (18.0 t/ha) compared to the sensitive genotypes (**Supplementary Table 6**). Accession 1666 and BDR Selection exhibited yield reductions of 29.2% and 31.4%, respectively, under the raised bed system relative to their control plots. However, when compared to the flatbed system under waterlogged conditions, the raised bed system increased yields by 40.6% for Accession 1666 and 41.3% for BDR Selection. In contrast, the sensitive genotypes suffered substantial yield losses, with reductions ranging from 41.6% to 64.8% under flatbed waterlogged conditions. Even in the raised bed system, these genotypes showed significant yield declines of 46.4% to 52.1% compared to their respective controls.

**Table 6:** Waterlogging tolerance indices of onion genotypes.

Genotype	Yp	Ys	TOL	MP	STI	SSI
Accession 1666	19.62	14.70	4.92	17.16	1.03	0.84
Accession 1630	17.50	13.32	4.18	15.41	0.84	0.80
W 355	15.53	11.88	3.65	13.71	0.66	0.79
BDR Selection	22.30	15.05	7.25	18.68	1.20	1.09
Bhima Red	15.83	9.57	6.27	12.70	0.54	1.32
Bhima Raj	15.57	10.25	5.32	12.91	0.57	1.14
Bhima Shubra	13.63	9.33	4.30	11.48	0.46	1.05
Bhima Super	13.67	9.57	4.10	11.62	0.47	1.00

Yp, Yield under control; Ys, Yield under stress; TOL, Tolerance level; MP, Mean productivity; STI, Stress tolerance index; SSI, Stress susceptibility index.

### Redundancy analysis

**Figure 3:**
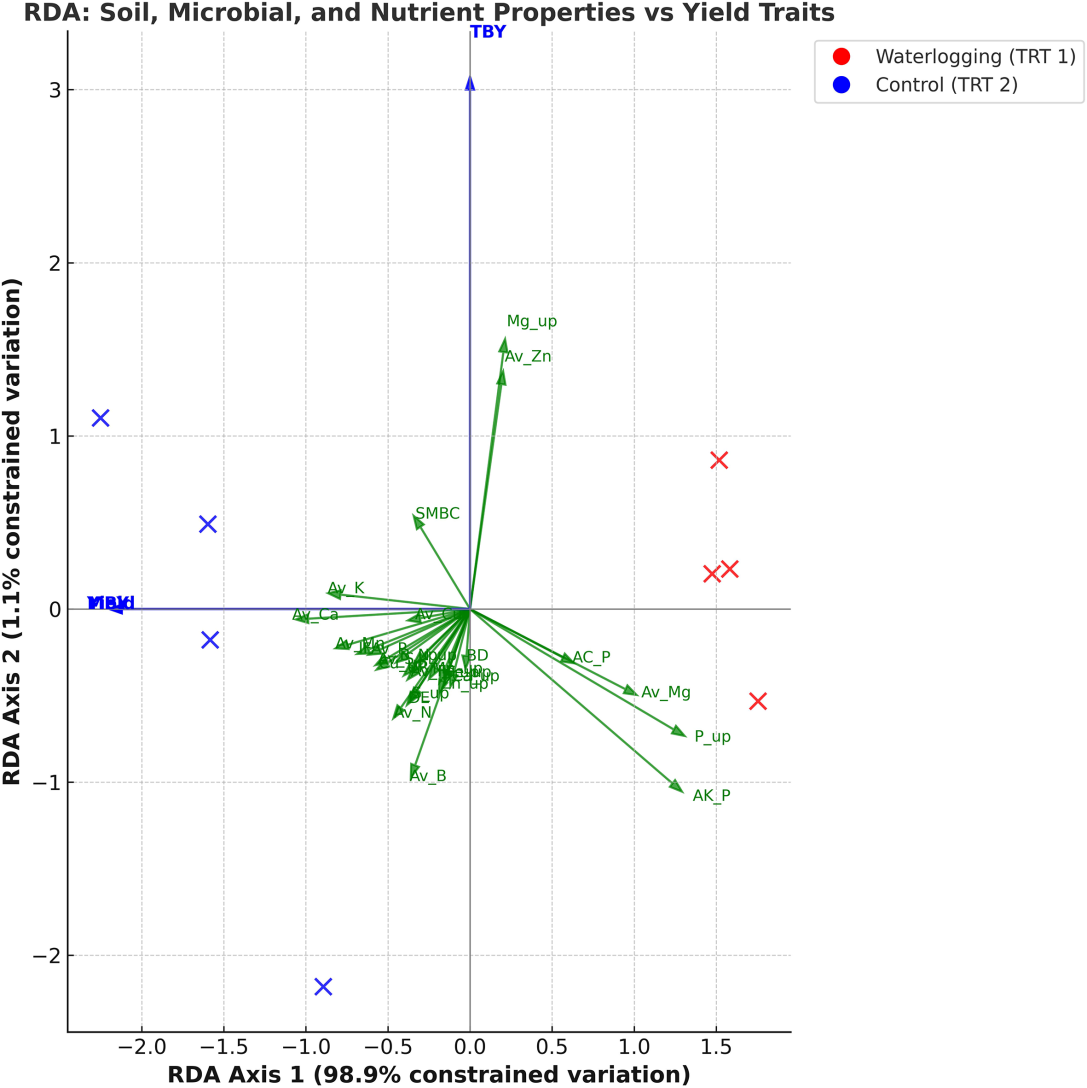
Redundancy analysis biplot illustrating the association of soil, microbial, and nutrient properties with onion yield under waterlogging and control conditions (n=8).

**Figure 4:**
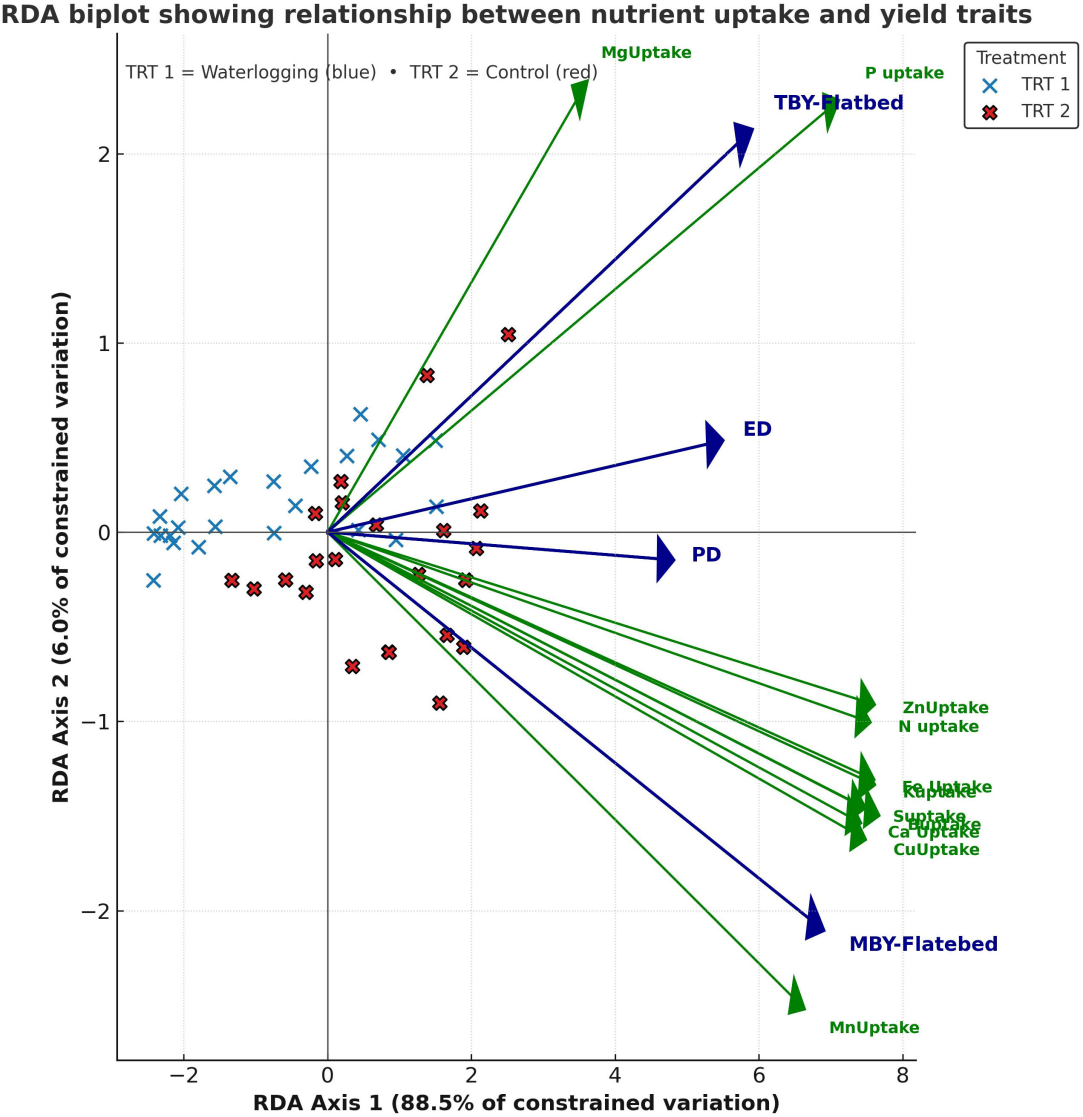
Redundancy analysis showing nutrient uptake–yield trait relationships under waterlogging and control conditions (n=64).

Exploratory RDA explained a total variation of 100%, with RDA1 accounting for 98.1% and RDA2 for 1.9% of the total variance. RDA1 distinctly separated waterlogged and control treatments, showing strong positive associations with infiltration rate and available macronutrients and micronutrients, as well as nutrient uptake and yield parameters, and a negative association with bulk density and acid phosphatase activity (**Figure 3**). RDA2 explained a smaller proportion of the variation, mainly associated with soil biological parameters. A separate RDA explaining 94.5% of the variation in nutrient uptake–yield relationships revealed that RDA1 (88.5%) clearly separated waterlogged and control treatments (**Figure 4**). Control plots showed strong association with N, P, K, Ca, Zn, and Cu uptake, along with higher bulb diameter and yield traits. RDA2 captured minor within-treatment variation, mainly linked to Mg and Mn uptake.

## Discussion

Rainfall plays a vital role in maintaining adequate water supply for domestic and agricultural needs (Biswas et al., 2025). A normal distribution of rainfall is crucial for groundwater recharge, domestic use, and sustaining crop productivity (Woldearegay et al., 2024). However, rainfall deviations—either deficient or excessive—can severely affect agricultural yields, especially for sensitive crops like onion (Thimmegowda et al., 2025). Although drought stress is often manageable through supplemental irrigation (Thangasamy et al., 2023), waterlogging—especially during the monsoon season—remains a more severe and complex challenge (Wakchaure et al., 2023). Prolonged soil saturation creates anaerobic conditions that limit oxygen availability (Gedam et al., 2022), alter microbial communities, and disrupt nutrient transformations, thereby impairing nutrient availability and plant uptake (Gedam et al., 2023). To address these effects, this field experiment examined the effect of waterlogging on soil physical, chemical, and biological properties, as well as nutrient availability, uptake, growth, and yield performance of tolerant and sensitive onion genotypes under waterlogged conditions in clay loam soils.

### Waterlogging effects on soil physical and microbial properties

Waterlogging increased bulk density and significantly reduced infiltration in the clay loam soil, primarily due to pore clogging by dispersed clay particles (Wu et al., 2017). A similar increase in bulk density, along with reduced infiltration rate, hydraulic conductivity, and permeability, as well as increased runoff, was observed by Kaur et al. (2019). The RDA biplot clearly separated control and waterlogged plots, with infiltration rate and soil available nutrients showing strong positive loadings and bulk density negative loadings, confirming that soil compaction and poor aeration were the principal factors influencing nutrient dynamics and yield variation.

Oxygen deficiency under waterlogging significantly affected soil biological properties (Manik et al., 2019). Reduced SMBC and dehydrogenase activity observed at 55 DAT in the present study indicated a shift in the microbial community from predominantly aerobic to anaerobic organisms (Tyagi et al., 2024), consistent with earlier findings by Ponnamperuma (1984) and Martínez-Arias et al. (2022). In this study, SMBC, enzyme activities, and nutrient availability together accounted for nearly all the explained variation, confirming the central role of microbes in nutrient transformation and mineralization processes. The strong positive correlation between microbial activity and nutrient availability under control conditions suggests that microbially mediated nutrient cycling was the key mechanism maintaining soil fertility. In contrast, reduced microbial efficiency under waterlogging led to declines in N and P mineralization and nutrient uptake, as also supported by the RDA results. These findings warrant further validation through multi-season and multi-location datasets to establish the robustness of these mechanisms.

### Redox effect on nutrient availability

Unlike studies with continuous submergence, our experimental design involved daytime-only flooding (8 AM to 6 PM), resulting in fluctuating redox conditions. These periodic oxidizing phases during the night may have contributed to limited microbial Fe and Mn reduction, leading to the re-oxidation and precipitation of Fe²^+^ and Mn²^+^ into unavailable forms. Additionally, repeated flooding and drainage cycles may have facilitated leaching/run off of micronutrients from the surface soil (Sao et al., 2023; Rupngam and Messiga, 2024). This may partially explain the observed decline in available Fe, Mn, Cu, and B in the present study. In contrast, a unique increase in Zn availability was observed under waterlogged conditions, possibly due to organic matter–mediated chelation and transient redox mobilization—an observation not previously reported in onions.

During the recovery phase (55–75 DAT), partial restoration of microbial activity and nutrient availability indicated redox stabilization (Wang et al., 2017; Rupngam et al., 2024). The post-waterlogging increase in micronutrient concentrations may be attributed to adaptive chemical equilibria that regulate and stabilize micronutrient solubility (Sathi et al., 2022). However, these nutrient levels remained lower than in control soils, reflecting incomplete microbial and chemical recovery and restricted oxygen diffusion in the fine-textured clay loam (Rupngam et al., 2024). The positive association among microbial biomass, nutrient uptake, and yield further confirmed the critical role of root–soil–microbe interactions in stress adaptation (Tyagi et al., 2024; Muhammad et al., 2024).

### Waterlogging effects on nutrient uptake, and plant growth and yield

Waterlogging significantly decreased both macro- and micronutrient uptake, as evidenced by the strong positive correlations observed between soil nutrient availability and plant uptake. This decline could be attributed to reduced soil aeration, altered nutrient solubility, and impaired root functions under waterlogged conditions (Zhang et al., 2025). However, tolerant genotypes like Accession 1666 and BDR Selection, which recorded higher stressed yields, exhibited higher STI and lower to moderate SSI values, indicating their superior performance and partial resilience under waterlogged conditions. Their relatively higher nutrient uptake under waterlogged conditions resulted in improved physiological adaptations such as enhanced aerenchyma formation, which enables internal oxygen transport to submerged roots (Pan et al., 2021; Zhang et al., 2025). The clear separation of treatments in the RDA biplot indicates that nutrient uptake and yield traits were strongly influenced by waterlogging. Under control conditions, enhanced uptake of N, P, K, S, and secondary and micronutrients (Ca, Mg, Fe, Zn, Cu, and B) contributed to improved metabolic processes, bulb size and yield, reflecting better root activity and translocation efficiency (Leon et al., 2021). In contrast, waterlogging markedly reduced nutrient uptake due to restricted root respiration and impaired active transport processes, leading to poor nutrient translocation and smaller bulbs. The strong positive associations of yield components (TBY, MBY, ED, and PD) with nutrient uptake confirm that nutrient uptake directly associated with bulb size and yield under normal control conditions. Elevated Ca, Mg, and S levels associated with bulb size indicate their role in maintaining bulb firmness, structural integrity, and quality under waterlogging stress. Although tolerant genotypes performed relatively better under waterlogged conditions, they still exhibited reduced growth and yield compared to controls.

Conversely, the weak or negative associations under waterlogging emphasize that oxygen deficiency disrupts rhizosphere nutrient dynamics, root metabolism, nutrient uptake and exhibited poor recovery (Ponnamperuma, 1984; Ma et al., 2022). This resulted in reduced leaf area, plant height, and biomass (Olorunwa et al., 2022). The decline in bulb size and yield across all genotypes confirmed waterlogging’s negative effects on onion growth and development (Thangasamy et al., 2023). However, the ability of tolerant genotypes to sustain dry matter and bulb yield through improved nutrient uptake and plant growth compared to sensitive genotypes in the present study highlight their suitability for waterlogged environments (Garcia et al., 2020). These findings highlight the potential of selecting genotypes with waterlogging stress tolerance mechanisms to support sustainable agricultural practices in areas increasingly affected by climate variability.

The flatbed system effectively simulated monsoonal waterlogging and enabled to identify onion genotypes capable of performing under waterlogged conditions. Since waterlogging is a major constraint during the monsoon, its negative effect can be effectively managed by integrating tolerant genotypes with raised bed planting methods (Thangasamy et al., 2023). In a parallel experiment, tolerant genotypes grown on raised beds produced higher yields than those on flatbeds under waterlogged conditions, indicating that this integrated approach has significant potential to enhance productivity in waterlogged environments (Manik et al., 2019). Although monsoon yields were lower overall, higher seasonal market prices and reduced labor requirements make monsoon onion cultivation economically viable when stress-tolerant genotypes are used.

## Conclusion

This study demonstrated that monsoonal waterlogging significantly altered soil physical and biological properties by increasing bulk density and reducing infiltration and microbial activity. These changes impaired nutrient availability and uptake, resulting in reduced growth, bulb size, and yield in onion crops. However, tolerant genotypes such as Accession 1666 and BDR Selection maintained higher nutrient uptake, produced higher yield, and showed enhanced recovery and adaptability under waterlogging stress than sensitive genotypes. Moreover, these genotypes achieved higher yields under raised-bed planting systems than in flatbed systems, indicating that integrating tolerant genotypes with raised-bed cultivation effectively reduces yield loss and enhances productivity under monsoon conditions. These findings highlight the potential of combining genetic tolerance with adaptive management practices to strengthen climate resilience and sustainability in waterlogging-prone environments. Future research will focus on establishing mechanistic linkages among soil physical properties, microbial activity, nutrient availability, nutrient uptake, and yield using larger and more representative sample sets. It will also aim to elucidate how waterlogging alters soil structure, nutrient dynamics, root anatomy, and microbial community composition under both flatbed and raised-bed systems, to comprehensively define the integrated soil–microbe–root–plant interactions governing onion tolerance to waterlogging.

## Data Availability

The onion genotypes used in this study are maintained at the ICAR-Directorate of Onion and Garlic Research (ICAR-DOGR), Pune. All authors have institutional authorization to use these materials for research purposes. Accession 1666 (IC645764; INGR22082) is a registered germplasm with the ICAR-National Bureau of Plant Genetic Resources (ICAR-NBPGR), New Delhi. Seeds of this accession have been deposited at both ICAR-DOGR and ICAR-NBPGR to ensure long-term conservation and availability. Voucher specimens for Accession 1666 and BDR selection have been deposited at ICAR-DOGR, and the material is publicly accessible upon request as per ICAR-NBPGR guidelines. Taxonomic identification as Allium cepa L. was confirmed by subject matter experts at ICAR-DOGR. The genotypes used in the present study were identified by Gedam et al. (2022), and their registration has been published (Gedam et al., 2024).
