# Supplementary material for: Effects of waterlogging on microbial activity, soil nutrient availability, nutrient uptake, and yield of tolerant and sensitive onion genotypes
Source: Front Plant Sci. 2025 Nov 13;16:1692450. doi: 10.3389/fpls.2025.1692450 (PMC12658594; doi:10.3389/fpls.2025.1692450)
Supplement: Supplementary file 2 [file Table2.docx]

Supplementary table 2: Correlation coefficient between plant nutrient uptake, plant growth parameters and bulb yield (n=64)

|  | PH | NL | TLA | TDY | Total N | Total P | Total K | Total B | Total Mn | Total Cu | Total Fe | Total S | Total Zn | Total Ca | Total Mg | Yield |
| --- | --- | --- | --- | --- | --- | --- | --- | --- | --- | --- | --- | --- | --- | --- | --- | --- |
| PH | 1 |  |  |  |  |  |  |  |  |  |  |  |  |  |  |  |
| NL | 0.72*** | 1 |  |  |  |  |  |  |  |  |  |  |  |  |  |  |
| TLA | -0.15* | -0.18* | 1 |  |  |  |  |  |  |  |  |  |  |  |  |  |
| TDY | 0.57*** | 0.21* | -0.21* | 1 |  |  |  |  |  |  |  |  |  |  |  |  |
| Total N | 0.58*** | 0.21* | -0.19* | 0.99*** | 1 |  |  |  |  |  |  |  |  |  |  |  |
| Total P | 0.59*** | 0.26** | -0.11 | 0.94*** | 0.94*** | 1 |  |  |  |  |  |  |  |  |  |  |
| Total K | 0.49*** | 0.31*** | -0.13 | 0.81*** | 0.79*** | 0.84*** | 1 |  |  |  |  |  |  |  |  |  |
| Total B | 0.60*** | 0.26** | -0.17* | 0.97*** | 0.97*** | 0.94*** | 0.85*** | 1 |  |  |  |  |  |  |  |  |
| Total Mn | 0.56*** | 0.30*** | -0.11 | 0.85*** | 0.86*** | 0.86*** | 0.90*** | 0.90*** | 1 |  |  |  |  |  |  |  |
| Total Cu | 0.55*** | 0.28*** | -0.18* | 0.92*** | 0.93*** | 0.88*** | 0.89*** | 0.94*** | 0.95*** | 1 |  |  |  |  |  |  |
| Total Fe | 0.47*** | 0.22** | -0.21* | 0.92*** | 0.91*** | 0.88*** | 0.91*** | 0.92*** | 0.92*** | 0.97*** | 1 |  |  |  |  |  |
| Total S | 0.61*** | 0.27*** | -0.13 | 0.95*** | 0.95*** | 0.95*** | 0.86*** | 0.97*** | 0.91*** | 0.91*** | 0.89*** | 1 |  |  |  |  |
| Total Zn | 0.61*** | 0.26** | -0.16 | 0.97*** | 0.97*** | 0.94*** | 0.83*** | 0.96*** | 0.89*** | 0.93*** | 0.91*** | 0.95*** | 1 |  |  |  |
| Total Ca | 0.53*** | 0.24** | -0.15 | 0.94*** | 0.94*** | 0.92*** | 0.89*** | 0.94*** | 0.94*** | 0.97*** | 0.97*** | 0.93*** | 0.94*** | 1 |  |  |
| Total Mg | 0.56*** | 0.24** | -0.18* | 0.95*** | 0.95*** | 0.90*** | 0.83*** | 0.97*** | 0.90*** | 0.93*** | 0.92*** | 0.94*** | 0.94*** | 0.94*** | 1 |  |
| Yield | 0.05 | -0.01 | -0.14 | 0.81*** | 0.80*** | 0.85*** | 0.67*** | 0.81*** | 0.81*** | 0.72*** | 0.77*** | 0.75*** | 0.73*** | 0.70*** | 0.79*** | 1 |

PH: Plant height, NL: Number of leaves, TLA: Total leaf area, TDY: Total dry matter yield, Yield: Bulb yield

*Significant at p≤0.05, **Significant at p≤0.001, and ***Significant at p≤0.0001

A strong and significant positive correlation between nutrient uptake and bulb yield was observed in both the overall dataset (*n* = 64) and the subset used for exploratory correlation analysis (*n* = 8).
