# Supplementary material for: Effects of waterlogging on microbial activity, soil nutrient availability, nutrient uptake, and yield of tolerant and sensitive onion genotypes
Source: Front Plant Sci. 2025 Nov 13;16:1692450. doi: 10.3389/fpls.2025.1692450 (PMC12658594; doi:10.3389/fpls.2025.1692450)
Supplement: Supplementary file 3 [file Table3.docx]

Supplementary Table 3. Effect of waterlogging stress on plant height (cm) of onion genotypes at different growth stages

| Genotypes | Control | Water-logging | Control | Water-logging | Control | Water-logging | Control | Water-logging |
| --- | --- | --- | --- | --- | --- | --- | --- | --- |
|  | 45 DAT | | 55 DAT | | 75 DAT | | 90 DAT | |
| Accession 1666 | 34.6 | 34.5 | 40.1 | 32.0 | 51.0 | 40.3 | 52.3 | 43.4 |
| Accession 1630 | 35.0 | 36.3 | 39.4 | 28.4 | 51.6 | 36.6 | 52.9 | 40.8 |
| W 355 | 34.7 | 37.0 | 39.8 | 28.8 | 51.3 | 35.5 | 52.6 | 40.3 |
| BDR Selection | 34.9 | 37.0 | 38.4 | 29.7 | 51.3 | 37.8 | 53.6 | 42.2 |
| Bhima Red | 35.6 | 37.0 | 36.1 | 24.5 | 48.2 | 29.1 | 51.6 | 31.8 |
| Bhima Raj | 35.6 | 36.3 | 40.0 | 24.9 | 50.5 | 32.1 | 52.9 | 34.7 |
| Bhima Shubra | 32.3 | 35.0 | 36.0 | 22.6 | 48.5 | 28.9 | 49.9 | 32.6 |
| Bhima Super | 34.4 | 37.8 | 35.4 | 23.1 | 51.3 | 30.4 | 52.1 | 33.4 |
| Tukey–Kramer HSD values (P<0.05) | | | | | | | | |
| Waterlogging (W) | 3.1 | | 5.3 | | 1.6 | | 0.9 | |
| Genotype (G) | 3.5 | | 2.3 | | 2.8 | | 3.3 | |
| W×G | 5.7 | | 3.7 | | 4.6 | | 3.4 | |

W: Waterlogging, G: Genotypes, DAT: Days after transplanting, HSD: Honestly significant difference, BDR: Bhima Dark Red
