# Supplementary material for: Effects of waterlogging on microbial activity, soil nutrient availability, nutrient uptake, and yield of tolerant and sensitive onion genotypes
Source: Front Plant Sci. 2025 Nov 13;16:1692450. doi: 10.3389/fpls.2025.1692450 (PMC12658594; doi:10.3389/fpls.2025.1692450)
Supplement: Supplementary file 4 [file Table4.docx]

Supplementary table 4. Effect of waterlogging stress on leaf numbers of onion genotypes at different growth stages

| Genotypes | Control | Water-logging | Control | Water-logging | Control | Water-logging | Control | Water-logging |
| --- | --- | --- | --- | --- | --- | --- | --- | --- |
|  | 45 DAT | | 55 DAT | | 75 DAT | | 90 DAT | |
| Accession 1666 | 6.7 | 6.6 | 8.2 | 5.1 | 7.6 | 6.6 | 8.0 | 5.1 |
| Accession 1630 | 6.9 | 7.1 | 7.8 | 3.4 | 8.1 | 5.1 | 7.8 | 5.0 |
| W 355 | 6.9 | 6.9 | 7.7 | 4.7 | 7.9 | 5.0 | 7.9 | 4.8 |
| BDR Selection | 7.0 | 6.4 | 7.7 | 5.0 | 8.2 | 5.2 | 8.0 | 4.4 |
| Bhima Red | 7.1 | 7.3 | 6.8 | 3.2 | 7.3 | 4.2 | 7.8 | 7.1 |
| Bhima Raj | 6.6 | 6.6 | 7.6 | 3.2 | 7.6 | 4.1 | 7.6 | 7.0 |
| Bhima Shubra | 7.0 | 7.1 | 8.1 | 3.1 | 8.1 | 3.6 | 8.1 | 6.9 |
| Bhima Super | 7.1 | 7.2 | 8.2 | 3.0 | 8.2 | 4.0 | 8.2 | 6.6 |
| Tukey–Kramer HSD values (P<0.05) | | | | | | | | |
| Waterlogging (W) | 0.6 | | 0.7 | | 0.1 | | 0.1 | |
| Genotypes (G) | 0.7 | | 0.6 | | 0.3 | | 0.3 | |
| W×G | 1.1 | | 1.0 | | 0.4 | | 0.3 | |

W: Waterlogging, G: Genotypes, DAT: Days after transplanting, HSD: Honestly significant difference, BDR: Bhima Dark Red
