# Supplementary material for: Effects of waterlogging on microbial activity, soil nutrient availability, nutrient uptake, and yield of tolerant and sensitive onion genotypes
Source: Front Plant Sci. 2025 Nov 13;16:1692450. doi: 10.3389/fpls.2025.1692450 (PMC12658594; doi:10.3389/fpls.2025.1692450)
Supplement: Supplementary file 5 [file Table5.docx]

Supplementary Table 5. Effect of waterlogging stress on total leaf area (cm^2^) of onion genotypes at different growth stages

| Genotypes | Control | Water-logging | Control | Water-logging | Control | Water-logging | Control | Water-logging |
| --- | --- | --- | --- | --- | --- | --- | --- | --- |
|  | 45 DAT | | 55 DAT | | 75 DAT | | 90 DAT | |
| Accession 1666 | 61.5 | 59.0 | 134.4 | 127.7 | 228.9 | 122.9 | 212.9 | 175.3 |
| Accession 1630 | 69.9 | 69.2 | 134.5 | 135.9 | 210.7 | 80.0 | 220.3 | 135.9 |
| W 355 | 70.0 | 64.2 | 139.8 | 137.3 | 206.4 | 108.3 | 216.8 | 122.7 |
| BDR Selection | 75.9 | 68.9 | 139.0 | 125.9 | 201.0 | 117.6 | 231.3 | 137.7 |
| Bhima Red | 64.3 | 68.9 | 139.0 | 138.6 | 158.9 | 54.3 | 166.3 | 84.4 |
| Bhima Raj | 67.4 | 52.3 | 135.7 | 134.8 | 207.6 | 60.1 | 215.5 | 92.2 |
| Bhima Shubra | 74.8 | 67.0 | 139.9 | 139.2 | 216.3 | 55.4 | 209.6 | 72.0 |
| Bhima Super | 65.2 | 67.6 | 137.3 | 136.6 | 221.1 | 56.9 | 198.5 | 81.1 |
| Tukey–Kramer HSD values (P<0.05) | | | | | | | | |
| Waterlogging (W) | 14.0 | | 51.6 | | 23.7 | | 13.8 | |
| Genotype (G) | 15.8 | | 22.1 | | 17.4 | | 20.1 | |
| W×G | 25.6 | | 34.3 | | 28.2 | | 24.9 | |

W: Waterlogging, G: Genotypes, DAT: Days after transplanting, HSD: Honestly significant difference, BDR: Bhima Dark Red
