# Supplementary material for: Effects of waterlogging on microbial activity, soil nutrient availability, nutrient uptake, and yield of tolerant and sensitive onion genotypes
Source: Front Plant Sci. 2025 Nov 13;16:1692450. doi: 10.3389/fpls.2025.1692450 (PMC12658594; doi:10.3389/fpls.2025.1692450)
Supplement: Supplementary file 6 [file Table6.docx]

Supplementary Table 6. Effect of waterlogging stress on onion genotypes grown on raised bed planting system

| Genotypes | Marketable yield (t ha^-1^) | | Total Yield (t ha^-1^) | |
| --- | --- | --- | --- | --- |
|  | Control | Waterlogging | Control | Waterlogging |
| Accession 1666 | 22.68 | 16.04 | 22.95 | 16.24 |
| Accession 1630 | 22.13 | 12.31 | 22.41 | 12.59 |
| W 355 | 19.53 | 10.74 | 20.01 | 11.02 |
| BDR Selection | 26.20 | 17.96 | 26.43 | 18.13 |
| Bhima Red | 19.97 | 10.41 | 20.58 | 11.03 |
| Bhima Raj | 20.08 | 9.56 | 20.88 | 10.22 |
| Bhima Shubra | 16.97 | 8.13 | 18.42 | 8.83 |
| Bhima Super | 17.24 | 8.96 | 17.87 | 9.44 |
